# De-escalation of head-and-neck cancer radiotherapy: balancing cure and side effects

**DOI:** 10.3389/fonc.2026.1798321

**Published:** 2026-04-10

**Authors:** Fréderic Duprez, Jean-François Daisne, Eleonore Longton, Sandra Nuyts

**Affiliations:** 1 Department of Radiation Oncology, Ghent University Hospital, Ghent, Belgium; 2 Faculty of Medicine and Health Sciences, Department Human Structure and Repair, Ghent University, Ghent, Belgium; 3 Vlaamse Werkgroep Hoofd-HalsTumoren (VWHHT), Ghent, Belgium; 4 Department of Radiation Oncology, Iridium Netwerk, Ziekenhuis aan de Stroom (ZAS), Antwerp, Belgium; 5 Department of Oncology, University of Antwerp, Edegem, Belgium; 6 Department of Imaging and Pathology, Catholic University of Leuven, Leuven, Belgium; 7 Institut de Recherche Expérimentale et Clinique (IREC), Center of Molecular Imaging, Radiotherapy and Oncology (MIRO), Université Catholique de Louvain, Brussels, Belgium; 8 Department of Radiation Oncology, Cliniques Universitaires Saint-Luc, Brussels, Belgium; 9 Laboratory of Experimental Radiotherapy, Department of Oncology, KU Leuven, Leuven, Belgium; 10 Department of Radiation Oncology, Leuven Cancer Institute, University Hospitals Leuven Institution, Leuven, Belgium

**Keywords:** de-escalation, elective neck, head-and-neck cancer, HPV-related oropharyngeal cancer, quality-of-life, proton therapy, adaptive radiotherapy

## Abstract

Potential treatment-related side effects of radiotherapy for head and neck squamous cell carcinoma are substantial and affect long-term quality-of-life. This narrative review discusses de-escalation strategies aimed at reducing side effects without compromising oncologic outcomes. Four categories of radiotherapy de-escalation strategies are discussed. First, elective neck dose- and volume de-escalation have shown very low rates of isolated regional recurrence in prospective and retrospective cohorts, however clinical benefits in unselected patient cohorts remain modest. Personalized dose and volume-de-escalation is currently under investigation, e.g. in risk-adapted elective neck volume-reduction based on lymphatic drainage mapping. Second, in HPV-related oropharyngeal cancer, unselected de-escalation strategies seemed to be inferior in terms of cure and survival, however risk-stratified approaches for post-operative radiotherapy or response-guided radiotherapy after neoadjuvant systemic therapy are promising, especially in intermediate-risk patients. Phase III randomized trials are currently recruiting patients in this matter. Third, adaptive radiotherapy can correct anatomical changes during treatment, but prospective trials are now needed to demonstrate its clinical benefits and safety in case of reducing volumes of targets. Fourth, proton therapy offers substantial dosimetric sparing and reduced acute toxicity in oropharynx, nasopharynx and nasal cavity and paranasal sinus cancer patients, however broad implementation is still limited by cost and access.

## Introduction

### Role and modalities of radiotherapy in head and neck squamous cell carcinoma

Of all patients with squamous cell carcinoma of the head and neck (HNSCC) that will be treated with curative intent, more than 80% will receive radiotherapy in the primary, the post-operative or the palliative setting. Depending on the site of the primary tumor, the T- and N-classification, the general condition and in the post-operative setting the presence of extranodal extension (ENE) in lymph node metastases and section margins, radiotherapy is combined with surgery and/or systemic agents (1). Cisplatin is the concomitant systemic agent of choice, resulting in an overall survival (OS) benefit of 8.9%, 8.1%, 5.4% and 4.0% in primary CRT for stage III-IVA oral cavity, oropharynx, larynx and hypopharyngeal HNSCC (2). The Radiation Therapy Oncology Group (RTOG) 9501 and European Organization for Research and Treatment of Cancer (EORTC) 22931 studies have set a clear role for chemoradiation (CRT) as standard post-operative treatment in patients with ENE and/or positive section margins since the mid-2000’s (3, 4). An updated analysis confirmed the OS benefit and less cancer-specific mortality after adjuvant CRT compared to adjuvant RT, however with more other cause mortality after CRT. This points to the delicate balance of offering adjuvant CRT to any patient with high-risk features for locoregional recurrence and patient-specific risk factors for adverse events (5).

High dose radiotherapy is planned and administered to the primary tumor and nodal gross tumor volume (GTVp and GTVn, respectively) expanded to a high-risk clinical target volume (CTV) or, in the postoperative setting, to the high-risk CTV. A 3–5 mm margin is added to obtain the planning target volume (PTV) for inter- and intra-fraction variability in daily positioning of the organs at risk (OAR). Concerning elective neck irradiation (ENI), an intermediate dose is delivered to cover microscopic spread in lymph nodes in the neck (except in cT1-2N0M0 glottic cancer). International guidelines were published for selecting and delineation the different target volumes with standardized definitions and site-specific delineation rules, incorporating data from several anatomical, clinical and anatomopathological studies (6–8). Their wide adoption led to improved consistency, accuracy and quality of radiotherapy for HNSCC. These guidelines cover the primary setting, the ENI and the OAR (5–8). Recently, guidelines for post-operative radiotherapy (PORT) in oral cavity cancer also have come available (9).

### Side effects of head-and-neck radiotherapy

Unlike the relatively good chances of local, regional and distant control of locoregionally advanced HNSCC using CRT, the rigorous implementation of international delineation guidelines and the use of modern imaging such as ^18^fluoro-fluoro-deoxyglucose positron emission tomography added to computed tomography (^18^F-FDG-PET-CT) and MRI, radiotherapy for HNSCC is still associated with significant acute and late side effects. The most important acute side effects are oral and oropharyngeal mucositis leading to acute dysphagia, necessity of feeding tubes and/or hospitalization. The most important late effects include dysphagia (leading to malnutrition), aspiration (leading to pneumonia) or xerostomia (leading to dental decay and loss of taste). These side effects result in reduced quality of life (QoL) and can occur or increase even years after treatment (10–12).

### Purpose

This narrative review will focus on following promising radiation modalities that may lead to less side effects in patients treated for HNSCC:

1. De-escalation of ENI
  a. Dose-de-escalation
  b. Volume-de-escalation
2. De-escalation in HPV-related oropharyngeal cancer
3. Adaptive radiotherapy
4. Proton therapy

## De-escalation of the elective neck

### The role of ENI

Since more than 5 decades, the standard dose prescription for ENI is 50 Gy in 2-Gy equivalents (further abbreviated as EQD2), empirically based on ancient patient cohorts and radiobiological models demonstrating adequate regional control (RC) for microscopic disease and safety for the spinal cord (13–16). This 2D-radiotherapy prescription used opposing latero-lateral beams up to around 44 Gy to the entire neck, followed by reduced fields of photon beams anteriorly from the vertebrae and electron beams for the posterior neck enabling to spare out the spinal cord as electron beams did not reach the spinal cord. This technique however induced higher skin and soft tissue dose as well as difficulties in matching the edges of the photon and electron beams leading to under- or overdosage.

Four major developments have been introduced in HNC radiotherapy since the 70’s.

1. The introduction of 3D-imaging to evaluate the neck (instead of merely palpation), with computed tomography (CT), magnetic resonance imaging (MRI) and ^18^F-FDG-PET-CT (17). Using these standard imaging procedures, less patients have undetected microscopical nodal involvement and more patients with truly positive neck nodes are diagnosed and treated correctly up to curative doses. This leads to fewer patients in which 50 Gy ENI is needed to achieve RC
2. Guidelines for the selection and delineation of the ENI volumes based on surgical experience, leading to more accurate and uniform treatments (6, 7).
3. The wide adoption of intensity-modulated radiotherapy (IMRT) that enables to homogeneously conform the dose to the targets while sparing OAR, coupled with an accurate 3D dose prediction by modern treatment planning system (18, 19).
4. The use of concomitant radiosensitizing chemotherapy in locoregionally advanced disease leading to an additive biological effect, also on occult nodal disease.

### Arguments in favor of dose- and volume de-escalation of the elective neck

Dose- and volume-de-escalation in ENI are hypothesized to result in less side effects as a lower dose is delivered to OAR such as (and not limited to) the parotid and submandibular glands, the pharyngeal constrictor muscles (PCM), larynx and thyroid gland. A planning study comparing 46 Gy with 30 Gy ENI in 16 patients with HPV-related oropharyngeal cancer demonstrated significantly lower doses to the PCM (52.8 *vs*. 48.9 Gy), larynx (35.3 *vs*. 30.5 Gy) and thyroid (44.9 Gy *vs*. 30.3 Gy), translating in lower normal tissue complication probabilities (NTCP) for xerostomia in the ipsilateral parotid gland (6.4% *vs*. 5.0%) and hypothyroidism (3.3 *vs*. 0.05%). Logically the effect was more pronounced in cN0 patients (20). ENI also contributes to carotid artery stenosis. The actuarial risk has been shown to be as high as 29% at 8 years in 366 treated patients and strongly correlated with smoking, hyperlipidemia, diabetes, coronary artery disease and peripheral artery disease, all of these frequent comorbidities in HNC patients (21).

Contraintuitively, lower ENI doses might also contribute to better disease control as ENI is associated with decreased immune responses in mice experiments with HNSCC-cell lines (22).

The question therefore arises if ENI dose- and volume-de-escalation could safely be done without compromising RC and leading to clinical benefits in terms of less side effects.

### Clinical trials of dose-de-escalation of the elective neck

**Tables 1**–**3** summarize the methodology and results of de-escalation of ENI trials. We identified 2 randomized and 2 single-arm prospective studies directly comparing standard ENI to a dose-de-escalated schedule.

**Table 1:** Overview of elective neck dose-de-escalation studies.

Trial (reference)	P/R	Phase	De-escalation strategy for the EN	Number	Sites	Setting	Primary endpoint	Secondary endpoints	Median follow-up (months)
UPGRADE (23)	P	III Non-inferiority	34 Gy *vs*. 44 Gy (EQD2)	196 *vs*. 99	OPC, LAR, HYP	Stage II-IVA, accelerated RT-trial without chemotherapy	Normalcy of diet and PSS-HN scores at 1Y	RC in EN at 2Y. Acute and late toxicity (< *vs*. > 90 days), QOL until 2Y, functional performance until 2Y, recurrence, survival	25
Belgian trial 1 (24, 25)	P	III Non-inferiority	40 Gy *vs*. 50 Gy (EQD2)	100 *vs*. 100	OC, OPC, LAR, HYP	Stage I-IVA Mixed primary RT and primary CRT	Dysphagia at 6 months	LC, RC, DC, LR, RR, DR, OS, DFS, DSS, neck stiffness, skin toxicity, salivary gland toxicity, mucosal integrity	91
INFIELD (32)	P	II	40 Gy	72 (non-randomized)	OPC, LAR	Stage I-IVA Mixed primary RT and primary CRT	Isolated regional recurrence risk at 2Y.	LC, DC, OS, PFS. Acute and late swallowing and skin toxicity. QOL using PROMs.	25
Wilmington, North Carolina (33)	P	II	36 Gy	54 (non-randomized)	OC, OPC, LAR, HYP	Stage III-IVA and stage II BOT; excluding bilateral pathologic nodes. Primary CRT (cisplatin 35 mg/m²,6–7 cycles)	Regional recurrence in 36 Gy region.	Survival, G3–5 toxicity and QOL using PROMs.	36
Memorial Sloan Kettering (35)	R	–	30 Gy	276	P16-positive OPC	Stage I-III (AJCC-8) Primary CRT	LRC at 2Y	DFS, DMFS, OS, QOL using PROMs.	26
Memorial Sloan Kettering (36)	R	–	40 Gy	73 (non-randomized)	LAR, HYP, p16-negative OPC	Stage III-IVA Primary CRT	Isolated regional recurrence in 40 Gy region.	LRC, DC, 2^nd^ primary cancer except prostate or skin, competing mortality in absence of locoregional recurrence	23

CRT, chemoradiotherapy; DC, distant control; DFS, disease-free survival; DMFS, distant metastases-free survival; DSS, disease-specific survival; EN, elective neck; EQD2, 2 Gy-biologically equivalent dose; HD, high dose region; HYP, hypopharynx; LAR, larynx; LC, local control; LRC, locoregional control; OC, oral cavity; OPC, oropharynx; OS, overall survival; P, prospective; PFS, progression-free survival; PROMs, patient-reported outcome measures; R, retrospective; RC, regional control; RT, radiotherapy; Y, year.

**Table 2:** Disease control in elective neck dose-de-escalation trials.

Trial (reference)	Arms	RC (all neck node recurrences)	Isolated regional recurrences	Regional recurrence topography	LC	DC	Actuarial survival
UPGRADE (23)	34 Gy *vs*. 44 Gy (EQD2)	At 2Y: 90% *vs*. 92%	3/196 *vs*. 1/99	In HD: 3/196 *vs*. 2/99 In EN: 9/196 *vs*. 4/99 Outside EN: 0	At 2Y: 81% *vs*. 85%	At 2Y: 91% *vs*. 91%	OS: at 2Y: 83% *vs*. 79% DFS: at 2Y: 73% *vs*. 78%
Dose de-escalation, Belgian trial (24, 25)	40 Gy *vs*. 50 Gy (EQD2)	At 2Y: 87% *vs*. 95% At 5Y: 86% *vs*. 92% (*p* = 0.10)	8/200; 6 (40 Gy) *vs*. 2 (50 Gy)	In HD: 7 *vs* 5 In EN: 2 *vs*. 1 Outside EN: 2 *vs*. 0	At 2Y: 85% *vs*. 87% At 5Y: 84% *vs*. 86% (*p* = 0.51)	At 2Y: 88% *vs*. 81% At 5Y: 87% *vs*. 76% (*p* = 0.07)	OS: at 2Y: 74% *vs*. 73%; at 5Y: 57% *vs*. 50% (*p* = 0.56) DFS: at 2Y: 61% *vs*. 62%; at 5Y: 48% *vs*. 41% (*p* = 0.69)
INFIELD (32)	Single arm	At 2Y: 90%	2/72 (both in HD, no IRR in EN)	In HD: 5/7 In EN: 2/7	At 2Y: 91%	At 2Y: 86%	OS: at 2Y: 89% DFS: at 2Y: 79%
Wilmington, North Carolina (33)	Single arm	98%	1/54 (persistent disease)	In HD: 1/1 In EN: 0	96%	96%	OS: at 3Y: 91%
Memorial Sloan Kettering (35)	Single arm	n.r.	5/276	In HD: 4/5 In EN: 1/5	n.r.	n.r.	OS: at 2Y: 95% DFS: at 2Y: 88%
Memorial Sloan Kettering (36)	Single arm	5/73	0	In HD: 5/5 In EN: 1/5 * Outside EN: 2/5 **	n.r.	At 2Y: 87%	n.r.

DC, distant control; DFS, disease-free survival; EN, elective neck; HD, high dose region; HR, hazard ratio; LC, local control; n.r., not reported; OS, overall survival; RC, regional control; Y, years.

*this patient is also present in the 5 patients “in HD”.

**1 patient also present in “in EN” (n = 1) and both patient synchronous recurrence in primary GTV.

**Table 3:** Toxicity reductions in elective neck dose-de-escalation trials.

Trial (reference)	Arms	Acute toxicity	Late toxicity and late QoL
UPGRADE (23)	34 Gy *vs*. 44 Gy (EQD2)	PEG-tube need: 10.7% *vs*. 19.2%; HR 0.56 (*p* = 0.046) G≥3 dermatitis: 6.6% *vs*. 7.1% (n.s.) G≥3 mucositis: 62.2% *vs*. 65.7% (n.s.)	G≥3 mucosal ulceration 4.8% *vs*. 4.6% (n.s.) G≥3 laryngeal chondronecrosis 1.1% *vs*. 2.3% n.s.) G≥3 mandibular osteonecrosis 1.0% *vs*. 1.1% (n.s.) Dry mouth related reduced QOL (EORTC HN35) up to 2Y: 46% *vs*. 60% (*p* = 0.025)
Dose de-escalation, Belgian trial (24, 25)	40 Gy *vs*. 50 Gy (EQD2)	Dysphagia G≥3 at 3 months: 2% (40 Gy) *vs*. 11% (50 Gy) (*p* = 0.03) Mucositis: n.s. Dermatitis: n.s. Weight loss: n.s. PEG-tube usage: n.s.	Xerostomia G≥1 at 6m: 69% *vs*. 86% (*p* = 0.01) Xerostomia G≥1 at 18m: 37% *vs*. 49% (*p* = 0.03) Xerostomia G≥1 at 12m and 24m: n.s. Dysphagia at 6,12,18,24m: n.s. Xerostomia G≥3 at 6,12,18,24m: n.s. Neck stiffness at 6,12,18,24m: n.s. Skin toxicity at 6,12,18,24m: n.s. Mucosal integrity at 6,12,18,24m: n.s.
INFIELD (32)	Single arm	Preventive PEG: 10% and reactive PEG 33% Dermatitis G0-1: 54%, G2: 42%, G3: 4%	9% PEG-dependent >3m after therapy
Wilmington, North Carolina (33)	Single arm	G3 dysphagia: 80% PEG: 81% (reactive *vs*. prophylactic n.r.) G3 mucositis: 41% G3 xerostomia: 13% G4-5: none	QOL: FACT-HN scores returned to base level by 6 months.
Memorial Sloan Kettering (35)	Single arm	PEG: 6% (all reactive)	n.r.
Memorial Sloan Kettering (36)	Single arm	n.r.	n.r.

G, grade; HR, hazard ratio; n.r., not reported; n.s., not significant; QoL, quality of life; Y, years.

The largest randomized trial is the Dutch UPGRADE-trial comparing ENI of 34 Gy EQD2 with 44 Gy EQD2 in a mixed population of stage II-IVA HNSCC. They randomized 196 *vs*. 99 patients, respectively (23). Patients were all treated with primary accelerated radiotherapy (66 Gy in 30 fractions over 5 weeks; after observing too much laryngeal toxicity 68 Gy in 34 fractions over 5.5 weeks) without chemotherapy. The dose planned to the elective neck was 43 Gy in 30 fractions, i.e. 34 Gy EQD2. Intermediate dose was prescribed to intermediate risk nodes (nodes with summed long- and short-axis diameter ≥17 mm and increased FDG-uptake). The trial demonstrated equal RC in both arms with 4.9% and 4.3% regional recurrences in the elective dose region in the de-escalated dose *vs*. the control arms, respectively. All patients that had recurrences in highly dosed pathologic nodes also had regional recurrences in the ENI field. It needs to be considered that the intermediate dose administered to the intermediate risk nodes (instead of administering purely ENI to these nodes) may have influenced RC. However, the primary endpoint of the study, normalcy of diet at 1 year, was not reached. The fact that there were almost no events of the primary endpoint measure in the control arm probably contributes to this.

A Belgian trial randomized 200 patients 1:1 in ENI to 40 Gy EQD2 *vs*. 50 Gy EQD2 in a mixed population of stage I-IVA HNSCC receiving primary radiotherapy or CRT. There was a trend toward more regional recurrences in the dose-reduced elective neck arm, but only 3 regional recurrences occurred in the elective neck (2 in the 40 Gy arm and 1 in the 50 Gy arm (24).There was no significant difference in acute toxicity during therapy but at 3 months after radiotherapy there was significantly less grade ≧̸3 dysphagia in the 40 Gy arm (25). Long-term toxicity profiles were better in the 40 Gy elective neck group with reduced dysphagia at 6 and 24 months and a lower incidence of any salivary gland toxicity (≧̸grade 1) at 6 and 18 months (26). QoL tended to be less impaired in the 40 Gy arm, but did not result in clinically relevant improvement. It was clear that merely lowering the elective dose to 40 Gy seemed not sufficient to reach better QoL-profiles (27).

A pooled analysis of 233 patients being treated with radiotherapy or CRT for stage I-IVA HNSCC with ENI of 40 Gy EQD2 was performed, including patients from 4 prospective trials: (1) patients treated in the above mentioned Belgian prospective trial (24), (2) patients from a consecutive phase-II randomized volume-de-escalation trial combined with adaptive radiotherapy (ART) (see hereunder for details) (28) and (3 and 4) 64 patients from 1 finalized and 1 recruiting ^18^F-FDG-PET-CT-based dose-escalation trial using 40 Gy for the elective neck (29, 30). With a median follow-up of 26 months, actuarial regional recurrence was 3.9%. Regional recurrences occurred in 28 patients, of which 12/28 were isolated regional recurrences. 9/28 patients had a regional recurrence in the ENI field *vs*. 21/28 in the high dose regions. 5/28 patients had their regional recurrence outside the elective neck, of which 1 patient presenting with a level I lymph node metastasis that was not treated as part of the experimental arm in the volume-de-escalation study (28). Only 1 patient had isolated regional recurrence in the elective neck treated with 40 Gy. 8 patients had regional recurrences combined with distant metastases or local recurrence (31).

Three non-randomized prospective trials were identified. The INFIELD trial included 72 patients with oropharyngeal and laryngeal cancer in a phase-2 study. Patients started with induction carboplatin-paclitaxel and received sequential radiotherapy with ENI of 40 Gy EQD2 (20 fractions of 2 Gy) to the elective neck in stage I-IVA HNSCC. There were no isolated regional recurrences in the dose de-escalated neck. There were 7 nodal failures, 5 of which were entirely within the 70 Gy volume and in 2 in the electively treated neck in combination with local recurrence (32).

A second prospective single-arm study included 54 patients with 36 Gy ENI for patients with stage III-IVA HNSCC excluding patients with bilateral adenopathies. One patient had persistent nodal disease but no elective regional recurrences were observed (33).

The phase II prospective DIREKHT trial combined dose- and volume-de-escalation in the primary tumor region as well as the elective neck after radical surgery for stage pT1–3 pN0-2b M0 HNSCC and is discussed in more detail in the chapter hereunder on volume-de-escalation (34).

Two retrospective studies were published from the Memorial Sloan Kettering Cancer Centre, New York (MSKC), that incorporated dose-de-escalated ENI in their clinical routine. They prescribed 30 Gy EQD2 ENI in locally advanced HPV-related oropharyngeal cancers. In selected negative neck patients, the retropharyngeal, level 1b and level V nodal basins were omitted from elective radiation. On 276 treated patients 5 isolated regional recurrences were observed, of which 1 in the elective 30 Gy region and 4 in the GTVn (of which 1/4 in a GTVn that was not identified as such before RT and treated up to 30 Gy, combined with local recurrence). Only three patients had a local recurrence in the high-dose PTV (35). In another retrospective study, the same research group reported the results of 73 patients with locally advanced laryngeal, hypopharyngeal, and p16-negative oropharyngeal cancers receiving a reduced elective neck dose of 40 Gy EQD2. There were no isolated neck recurrences. All 6 locoregional recurrences occurred in the 70 Gy region (36). A drawback of the latter study relies in the discretion of the physician for treatment of indeterminate nodes and the intermediate dose of 50–60 Gy around primary laryngeal and hypopharyngeal cancers introducing heterogeneity, especially as 7 centers were involved in this study for a limited number of 73 treated patients. The latter drawback however does not seem to impact on the purely elective neck dose of 40 Gy that was uniformly prescribed in all centers (36).

Although large variability in treatments, inclusion criteria, disease site distributions, stages, and numbers of p16-positive oropharyngeal cancers between the trials (**Table 2**), the total number of isolated regional recurrences in the dose-de-escalated elective neck for all trials is limited to 4/904 (<0.5%). The total number of regional recurrences including isolated and combined with local and/or distant recurrence is 22/904 (2.4%). These data as well as the data from all separate trials give confidence concerning oncological safety of de-escalating the ENI dose in the higher-risk patients.

### Clinical trials of volume-de-escalation of the elective neck

Consensus guidelines for selection of ENI volumes have led to uniform and safe treatments for all HNSCC patients (6). These guidelines are largely based on clinical series on RC. However, more patient-tailored selection of ENI volumes could be envisaged aiming at reducing the volumes of ENI. As an example, Moos et al. proposed a Bayesian network–based framework trained on a retrospective cohort of 428 patients treated for oropharyngeal cancer with definitive radiotherapy. By integrating clinical variables such as nodal spread patterns, T−stage, and tumor location, the model estimates ipsilateral and contralateral occult nodal risk. Application of predefined risk thresholds demonstrated substantial reductions in irradiated volumes while maintaining a low probability of missing subclinical disease, supporting the feasibility of risk-adapted elective irradiation (37).

**Tables 4**–**6** summarize the methodology and results of ENI volume-de-escalation trials.

**Table 4:** Overview of volume-de-escalation trials.

Trial (reference)	Phase	De-escalation strategy	Number	Sites	Setting	Primary endpoint	Secondary endpoints	Median follow-up (months)
DIREKTH (34)	II	Arm A: dose-de-escalation in primary tumor region in ≤pT2 without PNI/LVI and R>5mm. Arm B: no contralateral ENI in ≤3 ipsilateral pathologic lymph nodes with adequate contralateral sampling in non-lateralized tumors. Arm C: dose-de-escalation cf. arm A and ENI volume-de-escalation cf. arm B	150 A: 7 B: 95 C: 48	OC, OPC, LAR, HYP	All operated patients. Stage pT1-3, pN0-2B	LRC at 2Y	OS, DFS, late toxicity	36 months
INRT-AIR (38)	II	Non-randomized. No ENI Only treating involved nodes (70 Gy/35 fr) and suspicious nodes (66.5 Gy/35 fr) based on radiologic criteria combined with AI-based radiomics model	67	HPV-positive OPC (n=44) HPV-negative OPC (n=5) HYP/LAR (n=18)	Stage I-IVB Primary RT and primary CRT	Solitary elective volume recurrence	Patterns of failure PROMs	33 months
EVADER (39)	II	Non-randomized. Volume-reduced ENI based on low estimated occult nodal risk	103	HPV-positive OPC	cT1–3 N0–1 M0 cN1) Primary RT (n=53) or CRT (n=50, cisplatin).	EFS at 2Y	LRC Out-of-field RC, OS, toxicity, Patient-reported QoL	37 months
Belgian volume-de-escalation trial (28)	III Non-inferiority	Volume-reduced ENI based on low estimated occult nodal risk with ART *vs*. no reduced ENI volumes without ART. Multicenter trial, two centers, both centers used 20 x 2 Gy followed by sequential boost for high-risk targets	50 *vs*. 50	OC, OPC, LAR, HYP	Stage I-IVA Mixed primary RT and primary CRT	Acute and late (i.e.at 1Y) dysphagia	LC, RC, DC, tumor response at 3 months and acute and late (12 months) other toxicity	Minimally 1Y in all patients
Belgian sentinel trial (41)	I/II	ENI limited neck level containing the four most avid SLNs on SPECT/CT after 99mTc nanocolloid injection around the tumor	44	OC, OPC, HYP, LAR	Stage I-IVA Primary RT or CRT	2Y RF rate in the nonirradiated elective neck region	OS, dosimetry, NTCP, Patient-reported QoL	46 months

ART, adaptive radiotherapy; CRF, contralateral regional failure; CRT, chemoradiotherapy; DC, distant control; EFS, event-free survival; ENI, elective nodal irradiation; fr, fractions; G, grade; HPV, human papillomavirus; HYP, hypopharynx; LAR, larynx; LC, local control; LF, local failure; LRC, locoregional control; LVI, lymphovascular invasion; OC, oral cavity; OPC, oropharynx; OS, overall survival; PNI, perinereural invasion; PROMs, patient-reported outcome measures; QoL, quality of life; R, resection margin; RF, regional failure; RT, radiotherapy; SPECT/CT, single- photon emission computed tomography/computed tomography; Y, year.

**Table 5:** Results of elective neck volume-de-escalation trials.

Trial (reference)	Arms	RC (all neck node recurrences)	Isolated regional recurrences	Regional recurrence topography	Actuarial LC	Actuarial DC	Actuarial survival
DIREKTH (34)	All 3 arms	4/150	3	In HD: 0 In EN: 1* Outside EN (reduced volume): 4 (of which 1 patient **)	Only combined LRC reported: 96% at 3Y	At 3Y: 95%	OS: at 3Y: 94% DFS: at 3Y: 89%
INRT-AIR (38)	Single arm	At 2Y: 98% (OPC); 95% (LAR/HYP)	0	In HD: 1 In EN: 0 Outside EN: 1***	At 2Y: 96% (OPC); 79% (LAR/HYP)	At 2Y: 91% (OPC); 100% (LAR/HYP)	OS (OPC): at 2Y: 96% OS (LAR): at 2Y: 79% DFS (OPC): at 2Y: 90% DFS (LAR): at 2Y: 63%
EVADER (39)	Single arm	5/103 events of locoregional failure, not further specified	n.r.	In HD: n.r. In EN: n.r. Outside EN: 1	n.r.	n.r.	OS at 2Y: 95% DFS at 2Y: 92%
Belgian volume-de-escalation trial (28)	Volume-reduced ENI + ART *vs*. no reduced volume ENI, no ART.	At 1Y: 84% *vs*. 92% (*p* = 0.08)	5/100	In HD: 3/5 In EN: 1/5 (standard volume) Outside EN: 1 (reduced volume)	At 1Y: 86% (reduced volume) *vs*. 90% (*p*>0.05)	At 1Y: 82% (reduced volume) *vs*. 86% (*p*>0.05)	OS at 1Y: 74% (reduced volume) *vs*. 78% (*p*>0.05)
Belgian sentinel trial (41)	Single arm	At 2Y: 90%	1/44	In HD: 0 In EN: 3 Outside EN: 1	At 2Y: 88%	n.r.	OS at 2Y: 79%

ART, adaptive radiotherapy; DC, distant control; DFS, disease-free survival; ENI, elective neck irradiation; HYP, hypopharynx; LAR, larynx; LC, local control; n.r., not reported; OS, overall survival; RC, regional control; Y, years.

*in a patient with synchronous local and distant recurrence; **in a patient with synchronous local recurrence; ***in a patient with synchronous distant metastases

**Table 6:** Toxicity reductions of elective neck volume-de-escalation trials.

Trial (reference)	Arms	Acute toxicity	Late toxicity and late QoL
DIREKTH (34)			G≥2 dysphagia at 27m: 10% G≥2 dysphagia at 27m: 9%
INRT-AIR (38)	Single arm	G1 dermatitis: 90% G2 dermatitis: 10%	Composite MDADI improved at 12m (89) and 24m (92) compared to baseline (83) (*p<*0.05) PROMs
EVADER (39)	Single arm	G3/4 toxicities: 46%/1% %	5/103 local and/or regional recurrences 1/103 out-of-field RF
Belgian volume-de-escalation trial (28)	Volume-reduced ENI + ART *vs*. no reduced volume ENI, no ART.	G3 dysphagia, mucositis, dermatitis, pain, weight loss >10%, feeding tube dependency, hospitalization rate: n.s. (own data, not published)	G≥2 dysphagia at 12m: n.s. G≥2 xerostomia and neck fibrosis: n.s.
Belgian sentinel trial (41)	Single arm	n.r.	G1, 2, 3 xerostomia and dysphagia at 6m: unilateral *vs*. bilateral drainage and irradiation: n.s.

ART, adaptive radiotherapy; ENI, elective neck irradiation; G, grade; m, months; MDADI, MD Anderson Dysphagia Inventory; n.r., not reported; n.s., not significant; PROMs, patient-reported outcome measures.

The only randomized trial comes from the same Belgian research group as the previously described dose-de-escalation study and compared standard *vs*. reduced volume ENI was performed, with 40 Gy ENI in 100 patients, randomized 1:1 in non-adapted, non-ENI reduced volume radiotherapy *vs*. adaptive replanning for fractions 11–20 and 21–30 and reduced volume ENI for low risk elective neck volumes in the de-escalated arm. Regional recurrences were observed in 12/100 patients, 8 of which in the adaptive and volume reduced group *vs*. 4 in the control arm. There were 5 isolated regional recurrences, of which 3 in the GTVn region and only 2 isolated regional recurrences in the ENI field: 1 in the volume de-escalated arm outside the irradiated elective neck (that would have been treated in the control arm) and 1 in the control arm in the elective neck (28).

The most radical published trial in omitting ENI is the INRT-AIR prospective phase II study that evaluated the safety of entirely omitting ENI in patients with stage I-IVB HNSCC. Only involved and suspicious lymph nodes were delineated using radiologic criteria combined with AI-based radiomics model. Among 67 patients, no isolated regional recurrences were observed at two years, with limited toxicity, preserved swallowing function, and no chronic gastrostomy dependence in disease-free patients (38).

The Canadian EVADER-trial (CCTG HN.10) was a multicenter prospective phase II trial, evaluating volume-reduced ENI restricted to nodal levels with an estimated >5% risk of subclinical disease in low-risk HPV-related oropharyngeal HNSCC (T1–3 N0–1). Among 103 patients, 2-year event-free survival was 91.8%, with only one out-of-field regional failure and acceptable toxicity, while QoL largely returned to baseline by 24 months (39).

The already abovementioned prospective DIREKTH trial from Germany and Austria combined dose-de-escalation in the primary tumor region with volume-de-escalation in the elective neck after radical surgery for stage pT1–3 pN0-2b M0 HNSCC. This trial is of particular interest as it is the only published prospective ENI volume-de-escalation trial in the post-operative setting. A risk-adapted schedule was administered based on pathological risk factors. In low-risk patients for local recurrence (≤pT2, no lymphovascular or perineural invasion and resection ≥5 mm) the primary tumor region was dose-de-escalated to 56 Gy; in low-risk patients for regional recurrence (≤3 pathologic lymph nodes (in presence of a negative contralateral neck dissection with ≥6 examined lymph nodes in case of non-lateralized tumors) contralateral neck irradiation was omitted. In total 150 patients were included; primary tumor dose was reduced in 7 patients (5%; arm A); only elective neck volume reduction was done in 95 patients (63%; arm B) and combined elective neck volume and primary tumor dose reduction was done in 48 patients (32%, arm C. Cumulative incidence of locoregional recurrence was limited to 5.6% at 3 years: 4/143 ENI volume-de-escalated patients developed a nodal recurrence in the non-irradiated contralateral neck. Although also operated patient with laryngo-pharyngeal HNSCC were included most patients had oral cavity or oropharynx cancers, making firm conclusions on the laryngeo-pharyngeal cancer patients in this trial impossible (34).

A targeted and therefore promising strategy for volume-reduction of ENI uses sentinel lymph node (SLN) identification using single-photon emission computed tomography/computed tomography (SPECT/CT). Two prospective non-randomized studies demonstrated that lymphatic drainage mapping can safely guide selective ENI. The Dutch SUSPECT trial included 55 patients with lateralized HNSCC. Contralateral lymphatic drainage was observed in only 20%, allowing omission of contralateral ENI in a majority of patients. At median follow-up of 33 months, contralateral regional failure was only 2%, and SPECT/CT-guided ENI significantly reduced dysphagia, feeding tube dependence, and xerostomia and resulted in better QoL compared with a matched bilateral ENI group (40). A comparable approach was performed in a Belgian prospective phase I–II study in 44 cN0 HNSCC patients treated with definitive radiotherapy or CRT. Nearly half of patients had unilateral lymphatic drainage, allowing substantial reduction of ENI. After a median follow-up of 46 months, there was only 1 nodal recurrence occurring outside the SLN-guided target volume. Dose reductions to salivary glands, swallowing structures, and the thyroid translated into lower predicted toxicity and clinically meaningful improvements in QoL (41).

Several trials with reduced volumes of ENI are currently recruiting patients. Some of the most important ones are mentioned hereunder.

1. The Swiss multicenter phase-II DeEscO trial (NCT06563362) is enrolling 120 patients with clinically T1–4 N0–3 oropharynx cancer. Individualized ENI volume-reduction is done based on the results of a multi-institutional dataset of 598 patients on which a statistical lymphatic progression model incorporating tumor stage, nodal involvement, and midline extension was developed to estimate the risk of occult nodal metastases.
2. The Dutch SUSPECT-2 prospective one-arm trial (NCT03968679) is recruiting 90 patients with cT1-4N0-2b lateralized HNSCC. After SLN mapping procedure, dissection of SLN is performed in case of contralateral drainage. Contralateral ENI is performed only in case of pathologically proven metastases. The primary outcome is the rate of contralateral regional failure at 1 year
3. In the Belgian phase II SEMIRAHN trial, 147 HNSCC patients with unilateral nodal involvement will receive ipsilateral ENI at reduced EQD2, while contralateral treatment—delivered only if lymphatic drainage is detected—is randomized between the entire nodal level containing the draining SLN or the SLN alone, both at a reduced dose (NCT04688528).
4. The Canadian SELECT trial (CCTV HN11) is an international phase III non-inferiority study of 510 patients with lateralized oropharyngeal cancer, in which contralateral ENI will be administered only when drainage is observed. The primary endpoint is non-inferior disease-free survival compared with standard bilateral ENI, with secondary outcomes including QoL, swallowing function, xerostomia, and toxicity (NCT05451004).
5. The German/Austrian DIREKTH-2 trial is a prospective randomized phase II/III trial comparing standard postoperative volumes with or volume-reduction by eliminating ENI in pathologically non-affected nodal regions. In the phase II-part, 1-year PEG-tube dependence rate will be evaluated in 268 patients and if the primary endpoint is met, the phase III part will evaluate locoregional control in 508 patients, for both parts randomized 1:1 (NCT06030440).
6. The INVERT trial is a phase II prospective randomized trial in 80 patients comparing standard ENI *vs*. involved field ENI in stage I-IVB HNSCC with the assistance of diagnostic imaging and aided by an in-house developed AI-based algorithm to identify potentially cancerous nodes (NCT06477692).

### Concerns hampering wide-spread adoption of dose and volume-de-escalation in the neck

Although there are no arguments that dose or volume de-escalation in ENI would lead to more regional recurrences, de-escalated volume and/or dose prescriptions are generally not practiced in clinical routine. Several concerns retain radiation oncologists to incorporate a reduced dose to the elective neck in their routine practice. These concerns have been the subject of recently published eloquent reviews and editorials (42, 43). We tried to resume the concerns and hereunder.

1. Although ^18F^F-FDG-PET-CT improved diagnostic accuracy of pathologic neck lymph nodes compared to CT and MRI, its sensitivity and negative predictive value need to be higher to give confidence to radiation oncologists to de-escalate ENI. A systematic review reported pooled negative predictive values ranging between 81% and 96%, depending on the methodology used (17). However, related to the intrinsic characteristics of PET-detectors, major advancements are not to be expected in the near future.
2. Although planning studies and intuition raised high expectations for toxicity reduction in dose-de-escalation studies, evidence of clinical benefits in unselected patient cohorts remains limited. As an example, because of much better results than anticipated in the control arm, the UPGRADE-RT trial failed in demonstrating superiority in its primary endpoint, normalcy of diet at 1 year (23). First reason is that e.g. in this trial, high priority was given to xerostomia- and dysphagia-optimized dose planning in the benefit of patients in both study arms, concordant with the British DARS-trial, that demonstrated that radiotherapy-plan optimization without elective neck dose reduction improves patient-reported swallowing function compared with standard IMRT (19). Comparable efforts were made in most other de-escalation trials, e.g. the Belgian randomized dose-de-escalation trial that could only demonstrate modest improvements in grade≧̸3 dysphagia at 3 months, reduced dysphagia at 6 and 24 months and a lower incidence of any salivary gland toxicity (≧̸grade 1) at 6 and 18 months but there were no improvements in other toxicity outcomes or at other timepoints (24). Second reason relies in the concept of non-inferiority trials needing very high number of patients to detect small differences in side effects, which is clearly not easy to achieve in the heterogeneous group of HNSCC.
3. Even in the best-case scenario, regional recurrences are associated with invasive neck surgery and consequent morbidity. In the worst case they are associated with uncontrollable disease leading to major morbidity and eventually leading to death due to regional problems or distant spread. Radiation oncologists try to minimize this risk and mostly prefer 50 Gy ENI.
4. The heterogeneous nature of HNC in terms of site distribution, stage grouping, nodal burden, presence of ENE in positive neck nodes, prognostic factors in HPV-related *vs*. non-HPV-related oropharyngeal cancer and various combinations with systemic agents make that it is not entirely clear which subgroups are truly safe for de-escalation. It is possible that the reassuring results of the above-mentioned trials and cohort studies do not detect high-risk profiles for regional recurrence.
5. The setting of prospective trials is different from the setting of treatments in real-world clinical radiotherapy. De-escalation studies are performed after thorough staging using ^18F^F-FDG-PET-CT, mostly in intermediate/high-volume academic hospitals with stringent quality assurance, all of these favoring outcomes. All these characteristics are not necessarily present in routine clinical practice.
6. Although dose- and volume de-escalated ENI seem to be introduced in a limited number of radiation oncology centers (e.g. MSKCC), the absence of international consensus guidelines supporting ENI dose and/or volume-de-escalation clearly hampers its wide-spread use.

### Conclusions and recommendations

In conclusion and as pointed out previously by others (42, 43), several studies have demonstrated reassuring results with very few isolated regional recurrences after ENI dose-de-escalation leading to relatively modest benefits in term of acute and late side effects. However, these studies may be underpowered to detect small differences and are heterogeneous, hampering their wide-spread applicability.

Future studies should focus on toxicity: acute severe and late dysphagia with its complications, gastrostomy-use and xerostomia will be the most important side effects to tackle. Trials should be powered to detect the regional recurrence risk as well as meaningful QoL differences scored using patient reported outcome measures (PROMs). Risk-stratified de-escalation protocols, where elective dose and/or volume is tailored to individual patients based on tumor site, HPV-status, nodal risk, and other biomarkers as well as the patient’s individual preferences and general status should be studied. An alternative to such large randomized trials is to build real-life registries. For patients in specific subgroups, ENI dose- and or volume de-escalation might be more attractive. Patients with good prognosis such as stage I-II HNSCC and HPV-related oropharyngeal cancer and young patients that may survive for several decades may profit most from selective dose- and volume-de-escalation in regions at lower risk of occult nodal metastases given their better life expectancy. In such cases, and if well discussed with the patient, such a tailored ENI dose-de-escalation might be a valid option.

## De-escalation in HPV-related oropharyngeal cancer

Human papilloma virus (HPV)-related (mostly p16-positive) oropharyngeal squamous cell carcinoma (OPC) represents a distinct disease entity compared to non-HPV-related (mostly p16-negative) OPC, the latter being caused mainly by tobacco and alcohol use. A pivotal study of Ang et al. demonstrated superior disease control and survival in HPV-related OPC compared to non-HPV-related OPC (44) and this has been confirmed in many other studies. The incidence is steadily increasing in Western countries, including Flanders (45). The genetic differences underlying the carcinogenesis imply higher sensitivity to radiotherapy and chemotherapy, making these the preferred treatment options in locoregionally advanced tumors. Since prognosis is much better and patients are generally younger than patients with p16-negative OPC, mitigation of late side effects has been investigated in various de-escalation trials.

### Non-risk adapted de-escalation trials

The advent of minimally invasive surgery with transoral laser microsurgery (TLM) or transoral robotic surgery (TORS) provoked a revival of surgery in HNSCC. Less invasive surgery was assumed to be less toxic than radiotherapy. Different randomized studies have been conducted to compare TLM and/or TORS to primary CRT. The phase-2 ORATOR and ORATOR2 studies compared TLM/TORS with neck dissection and potential adjuvant CRT to definitive CRT (70 Gy in ORATOR and 60 Gy in ORATOR2) (46, 47). Against expectations, swallowing results at 1–2 years after treatment were better in the definitive CRT-arm. In ORATOR, the 5-year swallowing scores did not differ anymore between both arms; the 5-year OS and progression free survival (PFS) were 82-85% (47). Patients experienced more xerostomia in the radiotherapy arm while pain scores were worse in the surgery arm. The ORATOR2 trial was prematurely terminated after 61 included patients (on 140 expected) due to an excess of grade 5 toxicity in the surgery arm (46).

Other trials investigated de-escalation of CRT by replacing cisplatin by cetuximab (RTOG 1016, De-ESCALaTE, ARTSCAN III and TROG 12.01 studies) or reducing the total dose from 70 Gy to 60 Gy with 2 courses of cisplatin or replacing cisplatin by nivolumab in the (HN005 study) have been tested (48–52). All these randomized phase 3 studies have demonstrated the inferiority of de-escalation strategies while highlighting the importance of maintaining a high dose of radiotherapy and concomitant cisplatin.

All above-mentioned trials however included patients with HPV-related OPC largely based on the TNM7 classification without any further risk-adapted selection. Therefore, these trials might lack selection of risk-adapted patients that are most at risk of failure, irrespective of TNM staging. Selecting patients for personalized de-escalation (i.e. “risk-adapted”) prescriptions according to their individual prognosis is key. Until now, most published studies rely on neo-adjuvant treatments before radiotherapy, either with minimally invasive surgery or with systemic treatments.

### Risk-adapted de-escalation trials using minimally invasive surgery

The rationale for minimally invasive surgery using TLM or TORS is:

1. complete resection of low risk (LR) OPC (typically cT1–2 and cN0 or limited nodal metastases excluding ENE) that can avoid radiotherapy
2. de-escalation of the radiotherapy volume and/or dose while avoiding concomitant chemotherapy in intermediate-risk (IR) patients
3. de-escalation of the intensity of concomitant CRT in high-risk (HR) patients.

**Table 7** provides results of surgery-based radiotherapy de-escalation studies.

**Table 7:** Surgery-based selected de-escalation prospective studies in HPV-driven oropharyngeal carcinoma.

Trial (reference)	Phase	Tumor stage inclusion (TNM7)	N	Primary endpoint(s)	Secondary endpoints	Strategy	Results
E3311 (53, 54)	2	cT1–2 amenable to R0 resection No matted nodes on imaging	68	Feasibility 2Y PFS	Toxicity, swallowing, voice, head and neck symptoms, QoL	LR: no RT (arm A) IR: randomization between 50 (arm B) and 60 Gy (arm C) HR: 60–66 Gy + Cis_we_ (arm D)	4.5Y PFS, (arm): 93%(A), 95%(B), 90%(C), 86%(D) 4.5Y OS, (arm): 97%(A), 98%(B), 95%(C), 93%(D) Grade 3 mucositis and dysphagia: higher risk in arm D Lower QoL in arm C compared to arm D
AVOID (56)	2	pT1–2 without adverse features (no PNI, LVI, margins <2mm, R1)	60	2Y LC	2Y OS, LRFS, RRFS, DMFS, PFS	PT if reimbursed, XT otherwise No intentional RT to primary site RT to bilateral nodes only (54 to 66 Gy in 30–33 fractions)	PT for 45% of patients At 2Y: LC 98%; OS 100%; LRFS 98%, RRFS 98%; DMFS 96%; PFS 92% No feeding tube during RT
MCMS (55)	2	pT1–2 N1–3 without PNI, LVI, R1/2	61	2Y LC	2Y LRC, OS, PFS, DMFS, toxicity grade ≥3, QoL	PT if reimbursed, XT otherwise No intentional RT to primary site RT to ipsilateral or bilateral nodes only (54 to 60 Gy in 30 fractions)	PT for 72% of patients Ipsilateral only: 39% At 2Y (%): LC 98%; LRC 97%; OS 100%; PFS 95%; DMFS 98% 5 grade 3 dermatitis with PT
MC1273 (58)	2	Arm A (IR): pT3-4, pN2-3, LVI, PNI Arm B (HR): ENE	80	2Y LRC	2Y PFS, DMFS, OS, toxicity, swallowing, QoL	Arm A: 30 Gy in 1.5 Gy fractions twice per day + docetaxel 15mg/m2 on days 1 and 8; Arm B: 36 Gy in 1.8 Gy fractions twice per day + docetaxel 15mg/m2 on days 1 and 8	At 2Y: LRC 96% – Arm A 100%; Arm B 93%), PFS 91%; DMFS 95%; OS 99% Grade 3 toxicity: 16.5%. At 3 mo: 0%. QoL, swallowing, xerostomia: decreased direct after treatment, improved after 1Y.
MC1675 (57)	3	Cohort A (IR): pT3, pN2-3, LVI, PNI. Cohort B (HR): ENE.	194	Chronic cumulative grade ≥3 toxicity at 2Y	2Y DFS, OS, PFS, LRC, DMFS, QoL	Cohort A: Randomization: arm A of MC1273 (test arm) or 60 Gy (standard arm). Cohort B: Randomization: arm B of MC1273 (test arm) or 60 Gy + Cis_we_ (standard arm).	At 2Y (test *vs* standard arms): toxicity grade ≥3 (3% *vs* 11%), OS (97% *vs* 98%), PFS (88% *vs* 97%), DMFS (93% *vs* 98%). Prognosis worse in pN2 patients, particularly in test arm. QoL (xerostomia, pain, swallowing) significantly better in de-escalated arm, persistent after 2y.

DMFS, distant metastases free survival; DFS, disease-free survival; ENE, extranodal extension; HR, high risk; IR, intermediate risk; LR, low risk; LRFS, local recurrence free survival; LVI, lymphovascular invasion; n.s., not significant; OS, overall survival; PFS, progression-free survival; PNI, perineural invasion; PROMs, patient-reported outcome measures; PT, proton therapy; QoL, quality-of-life; RRFS, regional recurrence free survival; RT, radiotherapy; Y, year.

In the E3311 trial, 359 cT1–2 N0–2 patients were operated with TLM and neck dissection. Adjuvant treatment was administered according to the pathology-adapted risk group: none in LR (arm A), radiotherapy alone in IR-patients and high dose radiotherapy and weekly cisplatin in HR-patients (arm D). IR-patients were randomized between 50 (arm B) or 60 Gy (arm C). PFS at 544 months was 94 and 90% in arms B and C, respectively and overall survival (OS) 98 and 95%, respectively. PFS was 93% in arm A due to 4 late nodal recurrences, all in the 27 pN1 patients. This represents a crude rate of 14.8% which is at the edge of the 15% risk that is generally adopted to treat a volume to an elective dose, although caution in interpreting these numbers should be drawn given the limited number of pN1 patients in this trial (n=27) that makes definitive conclusions difficult. HR-features (> 1 mm ENE, R1 resection, > 4 positive nodes) identified correctly patients deemed for (standard) high dose CRT (53). As expected, grade ≥3 acute toxicity increased with treatment intensity. After an initial decrease in the RT arms, QoL returned progressively to baseline with time, although it was worse in arm C compared to arm B (54).

In cT1–2 cN1–3 tumors, a mucosal sparing strategy was tested in two prospective, single arm trials: the Alternative Volumes of Oropharyngeal Irradiation for De-intensification (AVOID) and the Mayo Clinic Mucosal Sparing (MCMS) trials (55, 56). After primary tumor resection and neck dissection, in absence of adverse features for local recurrence, adjuvant RT was administered to the nodal volumes while omitting entirely the oropharyngeal primary site. Proton therapy (PT) was administered in 45% and 72%, respectively. In the AVOID-trial, all patients received bilateral nodal radiotherapy, while in the MCMS-trial, 39% of the patients presenting well lateralized OPC received ipsilateral radiotherapy only. Concomitant chemotherapy was allowed for ENE in both trials. Oncological outcomes were excellent with 2Y-OS and local control (LC) of 100% and 98%, respectively. The mean non-intentional dose to the primary site was around 35 Gy in both trials and significantly lower using PT. No patient required tube feeding in the AVOID-trial (56). There was no swallowing impairment after radiotherapy in the MCMS-trial (55).

Even more pronounced de-escalation was tested in the phase-2, nonrandomized MC1273 trial, and in its follow-up phase-3, randomized MC1675 trial (57, 58). Risk-adapted doses of 30–36 Gy in 20 fractions of 1.5-1.8 Gy were administered twice daily with 2 weekly courses of concomitant docetaxel. In the MC1675 trial, it was compared to the standard-of-care (SOC) with concomitant weekly cisplatin for HR-patients. The rate of acute grade 3 toxicity was significantly lower in the test arm than in the SOC arm (3% *vs*. 11%) and its duration was shorter (2 versus 3 months). At 2 years, pain and swallowing were better in the de-escalated arm. OS ranged between 97 and 99%, however PFS was significantly lower in the de-escalated arm (81 versus 98%) and there was a trend towards a significantly lower distant metastases free survival (90 versus 97%, respectively). These differences were driven by distant metastases in the subgroup of patients with pathological ENE and pN2, illustrating the limits in treatment de-escalation in the more aggressive tumors (57).

### Risk-adapted de-escalation trials using neo-adjuvant systemic therapy

Various studies explored the possibility to de-escalate the radiotherapy dose according to the degree of response to neoadjuvant chemotherapy (59). Even if the oncological outcomes were encouraging, there were only phase 2 studies with limited number of patients and one phase 3 study terminated early due to poor accrual. Moreover, there was substantial variability in the neoadjuvant regimen, concomitant radiosensitization and dose prescription that was not related to the same degree of response. Last, the risk of failure remained higher in high-risk patients, as exemplified in the E1308 trial (60).

The only trials that adapted the loco-regional RT to both the risk and response are the phase 2 non-randomized OPTIMA and OPTIMA-II trials (61, 62). In OPTIMA, neo-adjuvant nab-paclitaxel and carboplatin were given for 3 cycles. In OPTIMA-II, nivolumab was added to the regimen. The radiotherapy dose and treatment intensity were adapted to the clinical risk (low versus high) and the Response Evaluation Criteria in Solid Tumors (RECIST)v.1.1 degree of response. Low-risk patients with a response of ≥50% received 50 Gy radiotherapy (or TORS in OPTIMA II); low-risk patients with 30-50% response or high-risk patients with a response of at least 50% received low dose RT (45–50 Gy) with concomitant chemotherapy; all the other patients received high-dose CRT. They enrolled 62 and 73 analyzable patients, respectively. The results were quite similar between both studies. In OPTIMA-II, the primary endpoint of adding nivolumab (+15% deep responses) was not reached: deep response (≥50%) was observed in 70% of the patients; about one third of patients were low-risk with a ≥50% response and a half were treated with low dose radiotherapy and concomitant chemotherapy. Overall, 2-years PFS and OS were ≥90% in both trials. However, the likelihood of grade 3 toxicity and of requiring a feeding tube decreased significantly in de-escalated treatment arms.

### Conclusions and perspectives for the next years

Until now, the non-risk adapted strategies have failed to demonstrate safety of de-escalation of therapy in patients with HPV-related oropharyngeal cancer. Even if HPV-related OPC is on average more radio- and chemosensitive than non-HPV-related OPC, the individual prognosis is heterogeneous. This was already recognized in 2010, when Ang et al. determined that smoking history also negatively impacts prognosis (44). Only the TROG 12.01 protocol excluded intermediate risk patients and the De-ESCALaTE study excluded patients with a smoking history ≤ 10 pack-years. Other biasing factors of the abovementioned trials are that in ORATOR 12% p16-negative OPC were included, and in the ARTSCAN III trial 27.5% p16-negative OPC or non-OPC. Meanwhile, TNM-classification version 9 included extranodal extension as crucial negative factor for prognosis in HPV-related OPC. Moreover, HPV-related OPC shows a lower mutational burden then HPV-negative OPC (63). As a consequence, immunogenic effect of cetuximab may be less pronounced, or even absent. In the IMCL-9815 trial, the benefit of cetuximab was not related to HPV-status (64), and in the EXTREME trial, the survival benefit was most pronounced in the p16-negative population (65). Last, but not least, the specificity of p16 immunohistochemistry alone is limited to 83%, implying a significant proportion of patients with a p16-positive OPC being HPV-non-related (66). If these patients received a de-escalated treatment in a trial, this would negatively alter the results of these studies. Of note, all patients in both OPTIMA trials had to have a mandatorily positive confirmatory HPV deoxyribonucleic (DNA) polymerase chain reduction (PCR) assay after p16 IHC positivity.

A risk-adapted selection strategy seems most promising in patients presenting a pathology-based intermediate-risk of recurrence after surgery (close margins, pN1 without ENE). It also highlights the importance of full intensity CRT for high-risk patients (R1, pN2 or ENE) (**Table 7**). After neo-adjuvant systemic therapy, both OPTIMA trials showed that a combination of clinical risk factors and degree of response would be another strategy to select patients for volume and dose de-escalation (61, 62).

These strategies are not ready yet for adoption in standard-of-care because of several reasons listed hereunder. However, promising developments are to be expected for the next upcoming years and might set new treatment paradigms in radiotherapy de-escalation for HPV-related oropharyngeal cancer.

1. The knowledge is largely based on phase 2 trials and only one phase 3 trial with limited number of patients (MC1675) (57). The results of the currently recruiting phase 2/3 “PATHOS” trial will be landmark for defining the next SOC in patients first operated with TORS or TLM+ND (67). A population of 1269 patients with cT1–3 N0-2b (TNM7) were recruited. Low-risk-patients will be just followed-up after TORS, intermediate-risk patients are randomized between 50 and 60 Gy and high-risk patients receive 60 Gy, randomized between concurrent weekly cisplatin or not. Study completion is expected by 2028 (NCT02215265).
2. The optimal prescription dose is largely unknown. Both MC1273 and MC1675 trials lowered the dose to 30–36 Gy in 2-daily fractions of 1.5-1.8 Gy over two weeks (57, 58). Since tumor cell repopulation would be negligible over such a short time, EQD2 would lay between 28.8-35.4 Gy. Hyperfractionated schedules are time-consuming and not handy for both the patient and the department. A once-daily fractionation schedule should be tested in a next trial. Dose-response curves should also be established in search of the optimal dose(s)/level(s).
3. Pathology stratification after surgery or degree of response after neo-adjuvant systemic therapy are imperfect clinical selection criteria. Biomarkers such as circulating tumor HPV-DNA (ctHPV-DNA) are now becoming available to better individualize the risk of recurrence. In the OPTIMA-II trial, 31 patients had paired samples (at baseline and after neo-adjuvant systemic therapy). Twenty-six patients had clearance while five patients still showed detectable ctHPV-DNA. PFS at 2 years was nearly 100% when ctHPV-DNA was undetectable against about 50% when detected (p=0.002) (61). The DART2 trial is a recruiting phase 2 trial incorporating ctHPV-DNA in combination with clinical and pathological factors after surgery, to better define the intensity of treatment (NCT05541016). Another possible biomarker is programmed death ligand-1 (PD-L1) expression that could predict response to neoadjuvant immunotherapy. Although the degree of response was not different between tumors with combined positive score (CPS) ≥20 or <20, the PFS tended to be improved if CPS was high in the OPTIMA II trial, making CPS a possible new target for patient selection (61). Anyway, the abovementioned biomarkers should not yet be used outside prospective validation trials.

## Adaptive radiotherapy

### Anatomical changes during radiotherapy

During the 6–7 weeks of fractionated radiotherapy for HNSCC, anatomical changes occur. OAR (e.g. the parotid glands) shrink and spatially migrate closer to the high-dose region, patients lose weight resulting in changed body contours that modify the radiation beam path and the primary tumor and pathologic lymph nodes can shrink (or in aggressive disease enlarge in the first weeks of treatment). These changes cause deviations between the planned *vs*. the delivered dose, mainly resulting in over-dosage to healthy tissues such as mucosa, swallowing muscles, skin, mandible and submandibular glands and parotids.

Adaptive radiotherapy (ART) refers to a single or several treatment adaptations during the 6–7 weeks radiotherapy course. Several factors predicting the need of ART in head-and-neck cancer have been identified and include bulky primary tumor and GTVn, larger volume parotid glands, larger body weight, primary nasopharyngeal cancer due to its vicinity to critical (neural) OAR and primary CRT without induction chemotherapy (68–70).

Historically ART needed a repeated CT-simulation and delineation which is resources and time-consuming, limiting its use at the discretion of the radiation oncologist in specific patients for whom adaptation cannot be neglected. Since some years, novel systems became available enabling daily treatment adaptations, i.e. so-called *on-line* ART (oART), using optimized CBCT-imaging or MR-based linear accelerators, coupled with automatic delineation and planning software.

### Dosimetric and clinical benefits of adaptive radiotherapy

A systematic review of 15 studies with at least one adaptive replanning for various types of HNSCC showed reductions in mean dose by 0.4–7.1 Gy for parotids and 0.5–5 Gy for spinal cord maximum dose (71). Many planning studies have been performed based on theoretical replanning based on daily imaging, but, only three clinical prospective randomized trials comparing toxicity or QoL of ART *vs*. non-adaptive radiotherapy for head-and-neck cancer have been published. **Table 8** gives an overview of published clinical trials investigating at least one planned treatment adaptation, excluding trials in which ART was part of a broader concept (e.g. dose-(de-)escalation) in order to rule disturbing effects.

**Table 8:** Prospective randomized trials comparing adaptive to non-adaptive radiotherapy.

Trial (reference)	Phase	Number treated with ART *vs*. non-ART	ART scheme	Sites	Setting	Primary endpoint	Secondary endpoints	Toxicity reductions	Trial remarks
ARTIX (72, 73)	III	64:63	Weekly off-line ART	OPC	Stage III-IVB	Xerostomia at 1Y	Salivary gland excretory function (scintigraphy); PROMs; early and late toxicity; OS, CSS, DFS Occurrence of second cancer. Cost data (Perrier 2024)	Xerostomia at 1Y 30% *vs*. 35% (n.s.)	Weekly off-line ART was not cost-effective in terms of reduction of xerostomia and better QOL compared with standard IMRT.
Maheshwari et al. (74)	Not specified	30:30	One treatment adaptation	All HNSCC	Stage III-IVB	No differentiation primary *vs*. secondary endpoints	No differentiation primary *vs*. secondary endpoints	G3 xerostomia at 6 months: 30% *vs*. 50% (*p* = 0.0149) No difference in G≥2 xerostomia	
DARTBOARD (75)	II	24:26	Daily ART	OPC, LAR, HYP	Stage I-IVB Mixed primary RT and primary CRT	Xerostomia at 1Y	PROMs: acute and late dysphagia and dermatitis. OS, PFS. LR, RR, DM.	G≥2 acute dermatitis: 8% *vs*. 31% (*p* = 0.01). No other significant differences	Reduced PTV-margins 1–2 mm in ART vs. 5 mm in non-ART.

ART, adaptive radiotherapy; CRT, chemoradiotherapy; CSS, cancer-specific survival; DM, distant metastases; DFS, disease-free survival; HYP, hypopharynx; LAR, larynx; LR, local recurrence; n.s., not significant; OPC, oropharynx; OS, overall survival; PFS, progression-free survival; PROMs, patient-reported outcome measures; RR, regional recurrence; Y, year.

1. The largest published trial was performed in France and randomized 127 patients in weekly off-line ART *vs*. non-adaptive radiotherapy. No difference was observed for the primary endpoint, xerostomia at 1 year. No other toxicity reduction was observed (72, 73).
2. In a trial from India, 60 patients were randomized between ART using one replanning at mid-treatment *vs*. non-ART. No differences in acute toxicity were seen. Late toxicity reduction was limited to less grade 3 xerostomia (30% in ART *vs*. 50% in non-ART, *p* = 0.0149) (74).
3. The first study using daily ART came from the University of Texas Southwestern Medical Center and has recently been published (DARTBOARD trial). They randomized a small cohort of 50 patients 24:26 in daily ART *vs*. non-ART. Taking into account the daily ART, reduced PTV-margins of 1–2 mm were used in ART *vs*. 5 mm in non-ART. There was less grade ≥2 acute dermatitis in the ART-group but no other significant differences (75).

Although innovative in their concept, the former two studies used physical 1- or 2-fold re-simulation without evaluating the potential benefits of daily ART. The latter study used daily ART but was limited in size, which could explain the limited observed toxicity. They showed that mean dosimetric differences do not translate automatically to clinical benefits for individual patients. This is largely due to the non-selection of patients benefitting the most from ART, based on the evolution of the NTCP for individual organs. There is a need for NTCP model-based randomized studies enriched with patients at significant functional benefit.

An appealing concept in toxicity mitigation using oART is the reduction of PTV-margins as has been done in the DARTBOARD trial (75). Classically, the intended dose is planned to the PTV to avoid marginal misses due to inter-fraction misalignment or intra-fraction movement. oART avoids inter-fraction misalignment, only leaving the possibility of intra-fractional motion. A classic PTV-margin using a thermoplastic mask and neck support for immobilization is an isotropic expansion of 3–5 mm from the high- and intermediate risk CTV. Also, for some important serial OAR, a planning-risk-volume (PRV) is created to avoid any risk of overdosage. In cases of close proximity to the GTV and CTV this can create overlap between PRV and PTV, resulting in underdosage to the PTV and thereby reduced tumor control probability. In any case, given the close proximity of OAR any high-dose volume reduction results in lower doses to OAR.

### Future perspectives for adaptive radiotherapy

Assessing clinical safety as well as objective reduction in side effects in prospective trials will be difficult, as argued above in the context of ENI dose de-escalation trials. Essentially, future studies should focus on the selection of the patients that will clinically benefit from ART, most evidently use of NTCP-modelling.

Another hurdle to tackle before oART for fractionated radiotherapy could be introduced in routine clinical radiotherapy is related to logistic and medico-legal issues. It can be expected that with the advent of novel treatment planning algorithms involving artificial intelligence, plan quality of automated plans will become better and be generated faster. However, adapted treatment plans still need to be approved by a radiation oncologist and physicist after critical revision, demanding important time input of human resources, especially in fractionated radiotherapy.

Given the practical, organizational and financial difficulties in generating evidence for novel technologies before even newer technologies become available, a proposed solution could be based on data collection through registries to build real-world evidence of its benefit. Importantly, safety in terms of disease control is mandatory before such new technologies should be offered to patients outside prospective trials in which patients are well-informed, specifically in case of reducing volumes of targets.

## Proton therapy

Proton therapy (PT) enables the delivery of high radiation doses to tumors while minimizing exposure to nearby OAR, owing to its characteristic Bragg peak and precise energy deposition within tissue. Preclinical and dosimetric studies have demonstrated that PT can significantly reduce OAR doses compared to intensity-modulated radiotherapy (IMRT), suggesting a potential for fewer acute and late toxicities (76, 77). Reduced radiation doses to critical structures such as the parotid glands, larynx, spinal cord, and brain have been consistently reported with PT.

However, many dosimetric studies do not fully account for setup uncertainties or anatomical changes during treatment, and it remains uncertain whether lower OAR doses translate into clinically meaningful benefits, particularly when both PT and IMRT achieve doses within accepted tolerance limits. Clinical evidence supporting PT in HNSCC therefore remains limited, with most data derived from retrospective, non-randomized, or single-institution studies. Consequently, identifying which patients are most likely to benefit from PT represents a major ongoing challenge as is also the case for the above-mentioned trials on ENI dose- and volume-de-escalation and radiotherapy de-escalation in HPV-related oropharyngeal cancer.

Currently, with roughly 100 PT centers operating worldwide, early clinical outcomes in HNSCC are beginning to emerge. Ongoing model-based selection strategies and prospective trials aim to clarify the true clinical value and cost-effectiveness of PT in this setting.

### Clinical evidence

PT is considered highly suitable for nasopharyngeal carcinoma (NPC) due to the complex anatomy and proximity of critical organs such as the optic nerves, brainstem, temporal lobes, pharyngeal muscles, and salivary glands. In patients treated with IMRT, 50–75% experience acute grade 3–4 toxicities, and 10–20% develop serious late effects (78, 79). Early clinical series of PT in NPC report reduced treatment-related toxicities, good compliance with combined chemotherapy, and excellent oncologic outcomes (80, 81). PT consistently lowers doses to the oral cavity, parotid glands, and mandible, translating to decreased rates of feeding tube placement, xerostomia, dysgeusia, and osteoradionecrosis.

Early reports suggest that PT can achieve similar disease control rates with less toxicity also in oropharyngeal cancer (82). In the largest retrospective study, moderate-to-severe xerostomia was significantly lower in the PT group at 18–24 months (6% *vs*. 20%; p = 0.025) and 24–36 months (6% *vs*. 20%; p = 0.01) (83). A pivotal Phase III randomized clinical trial (MDAC-study) compared intensity-modulated PT (IMPT) with intensity-modulated radiation therapy (IMRT) in patients with oropharyngeal cancer, of whom approximately 95% were HPV/p16-positive (84). The study enrolled 440 patients across 21 U.S. institutions, with a median follow-up of 3.2 years. The primary endpoint (PFS) was nearly identical between the two groups, with rates of 81.3% at 5 years for IMPT and 82.5% for IMRT, demonstrating non-inferiority of IMPT. OS rates after IMPT were 90.9% at 5 years versus 81% after IMRT (HR 0·58 [95% CI 0·34-0·99]; p=0·045). The nutritional status improved with IMPT. The cumulative incidences of gastrostomy-tube dependence at 60 days after the start of treatment were 26·8% (95% CI 20·5–34·6) for IMPT and 40·2% (32·4–49·1) for IMRT (p=0·018). This landmark trial is the largest of its kind to date and supports the integration of IMPT into standard care for OPC, particularly for patients with HPV-positive tumors.

The TORPEdO trial is a similar trial from the UK (85). Although IMPT achieved lower radiation doses to OAR, this did not translate into measurable clinical or patient-reported advantages at one year. Although TORPEdO and MDAC report similar findings in several respects—including comparable LC, regional and distant metastasis rates, and lower gastrostomy dependency with IMPT at the end of treatment (an absolute difference of 13% in both studies)—their conclusions differ significantly. This difference can largely be explained by the study design and the chosen evaluation timing. The TORPEdO trial primarily assesses the value of PT at one year post-therapy, while the MDAC study evaluates toxicity and clinical outcomes during treatment and up to ten years afterward. The risk of evaluating toxicity reduction at a time when toxicity is minimal (in the TORPEdO trial at 1 year) inevitably leads to an underestimation of the potential benefit of PT.

The role of PT in laryngeal/hypopharyngeal carcinoma is less investigated. In model-based selection studies (86), only a minority of patients with laryngeal SCC qualified for PT, indicating that the expected clinical benefit is smaller compared to nasopharyngeal or oropharyngeal sites.

The nasal cavity and paranasal sinuses are anatomically well-suited for PT, but these cancers are rare and histologically diverse, making direct comparisons with IMRT challenging. A meta-analysis of 41 observational studies reported that PT was associated with significantly higher loco-regional control than IMRT, with a median follow-up of 38 months (87). No difference in toxicity was reported between the 2 groups, aside from a higher rate of neurological complications in the PT group (20% of patients, compared to 4% in the photon group). However, there may have been reporting bias with a larger proportion of PT studies that reported treatment-related toxicities.

However, PT carries an increased risk of radiation-induced dermatitis (RD) due to the spread-out Bragg peak, with reported grade 3 RD rates ranging widely (35–100%) across studies (88–90). This variability likely reflects differences in institutional experience with PT planning. Despite this, many consider skin toxicity a manageable trade-off for reducing more severe, quality-of-life–impacting adverse effects. Overall, PT shows promise for achieving excellent tumor control while lowering long-term toxicities in HNSCC, especially in younger patients with HPV-related disease.

### Patient selection for proton therapy

As PT becomes more widely available, determining which patients benefit most from it has become increasingly important. Various institutions and research groups have proposed selection systems, but adoption in routine practice remains inconsistent. Early frameworks, like the Proton Priority System from the University of Pennsylvania, used weighted criteria (e.g., tumor site, stage, age, comorbidities) (91). Other models, such as those from Cheng et al. and the University of Adelaide, incorporated decision-support tools and simulation frameworks considering dosimetry, toxicity, cost-effectiveness, and quality-adjusted life expectancy (92, 93).

For adults, the model-based approach, first adopted nationally in the Netherlands, offers a more individualized and objective method. It uses normal tissue complication probability (NTCP) models to predict radiation side effects and compare photon versus proton plans. A patient qualifies for PT if the expected toxicity reduction (ΔNTCP) is clinically meaningful. This method has proven more cost-effective and adaptable, enabling personalized cancer care. Initial results from the University of Groningen showed that about 35% of eligible patients qualified for PT—mostly those with nasopharyngeal or oropharyngeal tumors (86). However, the current model includes only three NTCP outcomes (xerostomia, dysphagia, and tube feeding dependence) assessed six months post-treatment, meaning it may overlook other or long-term toxicities. To improve accuracy, patients are now included in data registries to expand models into comprehensive toxicity profiles (CITOR) (94).

Between 2018 and 2019, Tambas et al. reported the first clinical experience with model-based selection for PT (86). Of 227 patients referred for definitive radiotherapy, 172 (76%) were eligible for the selection procedure, and 80 (35%) ultimately qualified for PT. Most patients selected had mucosal squamous cell carcinoma (SCC) above the hyoid, including nasopharyngeal and oropharyngeal tumors, while only 12% of laryngeal SCC patients met the criteria.

The model-based selection is currently the most objective and individualized method for identifying HNSCC patients likely to benefit from PT. However, it depends on accurate NTCP modeling, consistent treatment planning quality, and ongoing refinement to include broader and longer-term toxicity data.

### Conclusion proton therapy

With the rise of HPV-related oropharyngeal cancer (OPC), which has a better prognosis, more long-term survivors now face late side effects such as dry mouth, swallowing difficulties and feeding-tube dependence. PT offers potential to reduce these toxicities. Reimbursement barriers remain a major obstacle since many eligible patients are denied proton therapy coverage, and funding shortages limit phase II/III trials. Global collaboration and unified reimbursement guidelines are therefore needed to increase access. One proposed solution is “coverage with evidence development”, where PT is reimbursed while data are continuously collected through registries to build real-world evidence of its benefit.

## General conclusion

Trials on dose and volume de-escalation, even with ENI doses of ≤40 Gy EQD2 and when omitting large volumes of elective neck, did not result in more isolated regional recurrences, the main point of concern in ENI de-escalation. However, the low incidence of isolated regional recurrences necessitate inclusion of high numbers of patients in randomized phase III trials to clearly provide evidence of non-inferiority. Most trials altogether included relatively low numbers of patients and not all of them were randomized. Moreover, reductions in side effects remain modest which is related to the nowadays use of xerostomia- and dysphagia-optimized IMRT-techniques. A more tailored approach for ENI de-escalation is based on SLN-mapping tailoring the ENI volume, currently performed in accruing studies.

Altogether, given the potentially devastating and sometimes fatal consequences of (isolated) regional recurrences, radiation oncologists remain reluctant to de-escalate the elective dose or volumes in routine clinics, however in specific cases, it might be envisaged if well discussed with the patient. Innovative techniques include sentinel node mapping enable more risk-adapted volume de-escalation and are currently under investigation.

Risk-adapted strategies in HPV-related oropharyngeal cancer include dose-de-escalation in post-operative radiotherapy based on surgical findings. The first trials applying such risk-adapted strategies are reassuring concerning oncologic safety.

Innovative radiotherapy techniques that become increasingly available include PT and oART. PT trials concentrated on nasopharyngeal, oropharyngeal and sinonasal cancer and demonstrated the oncologic safety. oART is a novel technique with a limited number of prospective trials. Patient-tailored approaches such as NTCP-based selection for PT as well as oART are necessary to avoid wide-spread use of sometimes futile and expensive techniques. Reimbursement and availability remain important problems for larger phase-III trials in expensive and rapidly evolving technical advancements. “Cost coverage with evidence development” is a proposed solution to generate real-world evidence by continuously collecting data through registries.
